# Editorial: Cerebrospinal fluid dynamics and intracranial pressure elevation–Novel insights on molecular and physiological mechanisms, and implications for neurological disease

**DOI:** 10.3389/fnmol.2022.1119980

**Published:** 2022-12-28

**Authors:** Adjanie Patabendige, Vegard Vinje, Marcus Stoodley

**Affiliations:** ^1^Brain Barriers Research Group, Department of Biology, Edge Hill University, Ormskirk, United Kingdom; ^2^The School of Biomedical Sciences and Pharmacy, The University of Newcastle, Newcastle, NSW, Australia; ^3^Department of Clinical Infection, Microbiology & Immunology, Institute of Infection, Veterinary, and Ecological Sciences, University of Liverpool, Liverpool, United Kingdom; ^4^Simula Research Laboratory, Department of Numerical Analysis and Scientific Computing, Oslo, Norway; ^5^Neurosurgery Unit, Faculty of Medicine, Health and Human Sciences, Macquarie University, Sydney, NSW, Australia

**Keywords:** cerebrospinal fluid, intracranial pressure, ischaemic stroke, hydrocephalus, idiopathic intracranial hypertension, neurovascular unit

Cerebrospinal fluid (CSF) provides a protective cushioning to the brain and the spinal cord, and serves an important role in nutrient transport and waste removal, essential for maintaining normal neuronal function. Homeostatic feedback mechanisms are responsible for the strict regulation of CSF formation, composition, volume, turnover, and flow (Bothwell et al., 2019). Dysregulation of these homeostatic feedback mechanisms during certain neurological diseases such as stroke, brain trauma, hydrocephalus and idiopathic intracranial hypertension (IIH) can cause CSF volume to rise. This can be life threatening, as it leads to an increase in intracranial pressure (ICP). Control of ICP is achieved *via* highly invasive procedures. Development of non-invasive treatments such as pharmacological agents is hampered by a lack of suitable models and incomplete understanding of the underlying pathophysiology. This Research Topic aimed to collect emerging evidence and novel perspectives on CSF dynamics and ICP elevation in neurological disease to highlight new developments in this field.

ICP elevation due to an increase in fluid build-up in the skull is experienced by patients with large malignant strokes, but this is not usually reported in minor-moderate strokes due to the need for highly invasive ICP measurement methods in the clinic (Helbok et al., 2014). Therefore, evidence for ICP elevation and underlying mechanisms in these strokes is mainly derived from pre-clinical stroke models (Omileke et al., 2021a,b). To further understand these mechanisms, Bothwell et al. explored cranial CSF clearance at cervical lymphatics using a tracer method to determine CSF movement into deep cervical lymph nodes and transit to the spinal subarachnoid space in a rat model of ischaemic stroke. The data suggested that a reduction in the CSF clearance through the cervical lymphatics may contribute to ICP elevation post ischaemic stroke. This was partially compensated by an increase in spinal CSF outflow as demonstrated by an increase in CSF transit to the spinal subarachnoid space. These findings could explain other studies which have reported increased resistance to CSF outflow post-stroke (Patabendige et al., 2019; Alshuhri et al., 2020), which indicate that a reduction in cranial CSF clearance is responsible for increased CSF outflow resistance in ischaemic stroke. According to the Monro–Kellie doctrine, the total volume of brain, blood and CSF is constant, and an increase in one of these components will cause a decrease in one or both of the other two components to maintain physiological ICP (Wilson, 2016). In their study of CSF micro-volume changes inside the spinal space, Klarica et al. introduced a new hypothesis that suggests an increase or decrease in ICP can occur due to small changes in spinal CSF volume without significant changes in CSF volume in the cranium. Their research points to a crucial role of the spinal subarachnoid space in controlling CSF pressure-that the spinal subarachnoid space plays a greater role than the intracranial space in compensating ICP fluctuations. However, others have also pointed towards the importance of considering the effect of epidural blood flow on the spinal pressures to fully understand the dynamics of CSF flow, especially during respiration (Lloyd et al., 2020).

Traditional views of CSF dynamics and ICP continue to be challenged as the field continues to evolve and new theories are formed, and novel models developed to study these complex mechanisms. Indeed, Eide and Hansson have provided a new perspective on the pathophysiology of IIH. While the pathophysiology of IIH is unknown, the authors challenged conventional studies that focus solely on intracranial CSF and venous pressures as main culprits. Their findings demonstrate that the majority of the patients included in their study had abnormal structural changes at the glia-neuro-vascular interface, suggesting capillary damage and blood-brain barrier (BBB) dysfunction as primary events in the pathophysiology of IIH. This suggests that BBB dysfunction and astrogliosis (Patabendige et al., 2021) could lead to an increase in brain volume and affect intracranial compliance, causing elevated ICP in IIH patients, while venous compression and increased CSF pressure may be secondary events. Furthermore, in their review, Yang et al. discuss the role of astrocytes in the pathophysiology of hydrocephalus, and stress the need for further studies, because astrocytes have not received the attention they deserve given their potentially important role in the pathophysiology of hydrocephalus. Others have demonstrated that “tissue compliance” could be an important factor, where neurons may also adaptively regulate their volume in diseases such as stroke in response to increased ICP, challenging the current dogma in pressure-volume relationships in neurological disease (Kalisvaart et al., 2020). A comprehensive review by Zhao et al. highlights the role played by aquaporins (AQP), which are highly expressed in astrocytic endfeet (AQP4) and choroid plexus epithelial cells (AQP1), in the pathophysiology of idiopathic normal pressure hydrocephalus, and outlined new targets for further research. Overall these studies and reviews demonstrate the important role played by the cells of the neurovascular unit in the pathophysiology of these diseases, and the need for further studies to determine their contribution to disease pathology. Other reviews in this topic discuss the role of the choroid plexus epithelium in neurological disease (Liu et al.) and the role of ependymal cilia in hydrocephalus (Ji et al.).

Finally, Hornkjøl et al. and Perera et al. have presented novel findings on CSF dynamics from a computational model and a non-invasive magnetic resonance imaging (MRI) technique respectively. Hornkjøl et al. demonstrated the importance of CSF dynamics on transport and clearance of substances, not only in the spinal subarachnoid space, but also deep into the brain. Research on the ‘glymphatic system’, a proposed waste clearance system of the brain, has mainly focused on fluid flow and transport within the brain (Iliff et al., 2012), while Hornkjøl et al. show that CSF flow in the spinal subarachnoid space may reduce characteristic clearance times of substances in the human brain from years to a few days. Perera et al. demonstrated the first ever application of a novel blood-cerebrospinal fluid barrier arterial spin labeling (BCSFB-ASL) MRI approach to determine BCSFB function in healthy and hypertensive rats. The findings suggest that BCSFB could be a key site vulnerable to systemic hypertension, leading to changes in CSF homeostasis in hypertensive subjects.

Overall, this Research Topic provides a cross-cutting perspective of CSF dynamics and ICP elevation, and showcases a range of thought-provoking approaches and models to study the complex underlying mechanisms. The novel discoveries presented here will be exciting to follow as the field moves forward from traditional concepts of CSF dynamics.

## Author contributions

Conceptualization and writing—original draft: AP. Writing—review and editing: AP, VV, and MS. All authors approved the submitted version.

## References

[B1] Alshuhri M. S. Gallagher L. McCabe C. Holmes W. M. (2020). Change in CSF dynamics responsible for ICP elevation after ischemic stroke in rats: a new mechanism for unexplained END? Transl Stroke Res. 11, 310–318. 10.1007/s12975-019-00719-6 31418164

[B2] Bothwell S. W. Janigro D. Patabendige A. (2019). Cerebrospinal fluid dynamics and intracranial pressure elevation in neurological diseases. Fluids Barriers CNS. 16, 9. 10.1186/s12987-019-0129-6 30967147 PMC6456952

[B3] Helbok R. Olson D. M. Roux P. D. L. Vespa P. The Participants in the International Multidisciplinary Consensus Conference on Multimodality Monitoring. (2014). Intracranial pressure and cerebral perfusion pressure monitoring in non-TBI patients: special considerations. Neurocrit Care. 21, S85–94. 10.1007/s12028-014-0040-6 25208677

[B4] Iliff J. J. Wang M. Liao Y. Plogg B. A. Peng W. Gundersen G. A.. (2012). A paravascular pathway facilitates CSF flow through the brain parenchyma and the clearance of interstitial solutes, including amyloid β. Sci Transl Med. 4, 147ra111. 10.1126/scitranslmed.3003748 22896675 PMC3551275

[B5] Kalisvaart A. C. J. Wilkinson C. M. Gu S. Kung T. F. C. Yager J. Winship I. R.. (2020). An update to the Monro-Kellie doctrine to reflect tissue compliance after severe ischemic and hemorrhagic stroke. Sci Rep. 10, 22013. 10.1038/s41598-020-78880-4 33328490 PMC7745016

[B6] Lloyd R. A. Butler J. E. Gandevia S. C. Ball I. K. Toson B. Stoodley M. A.. (2020). Respiratory cerebrospinal fluid flow is driven by the thoracic and lumbar spinal pressures. J Physiol. 598, 5789–5805. 10.1113/JP279458 32990956

[B7] Omileke D. Bothwell S. W. Pepperall D. Beard D. J. Coupland K. Patabendige A.. (2021a). Decreased intracranial pressure elevation and cerebrospinal fluid outflow resistance: a potential mechanism of hypothermia cerebroprotection following experimental stroke. Brain Sci. 11, 1589. 10.3390/brainsci11121589 34942890 PMC8699790

[B8] Omileke D. Pepperall D. Bothwell S. W. Mackovski N. Azarpeykan S. Beard J.. (2021b). Ultra-short duration hypothermia prevents intracranial pressure elevation following ischaemic stroke in rats. Front. Neurol. 12, 684353. 10.3389/fneur.2021.684353 34616350 PMC8488292

[B9] Patabendige A. MacKovski N. Pepperall D. Hood R. Spartt N. (2019). Correction to: A26 Cerebrospinal fluid outflow resistance is increased following small-moderate ischaemic stroke. Fluids and Barriers of the CNS. 16, 22. 10.1186/s12987-019-0144-7

[B10] Patabendige A. Singh A. Jenkins S. Sen J. Chen R. (2021). Astrocyte activation in neurovascular damage and repair following ischaemic stroke. Int. J. Mol. Sci. 22, 4280. 10.3390/ijms22084280 33924191 PMC8074612

[B11] Wilson M. H. (2016). Monro-Kellie 2.0: The dynamic vascular and venous pathophysiological components of intracranial pressure. J. Cereb. Blood Flow. Metab. 36, 1338–1350. 10.1177/0271678X16648711 27174995 PMC4971608

